# Keratin-based topical cream for radiation dermatitis during head and neck radiotherapy: a randomised, open-label pilot study

**DOI:** 10.1017/s1460396924000037

**Published:** 2024-04-15

**Authors:** Ryan T Hughes, Beverly J Levine, Bart A Frizzell, Kathryn M Greven, Mercedes Porosnicu, Thomas W Lycan, Luke R Burnett, Karen M Winkfield

**Affiliations:** 1 Department of Radiation Oncology, Wake Forest University School of Medicine, Winston Salem, NC, USA; 2 Department of Social Sciences and Health Policy, Wake Forest University School of Medicine, Winston Salem, NC, USA; 3 Department of Internal Medicine-Section of Hematology and Oncology, Wake Forest University School of Medicine, Winston Salem, NC, USA; 4 KeraNetics, Inc., Winston Salem, NC, USA; 5 Department of Radiation Oncology, Vanderbilt University Medical Center, Nashville, TN, USA; 6 Meharry-Vanderbilt Alliance, Vanderbilt University Medical Center, Nashville, TN, USA

**Keywords:** Head and neck cancer, patient-reported outcomes, radiation-induced dermatitis, radiation injury, radiodermatitis

## Abstract

**Introduction:**

Radiation dermatitis (RD) is a frequent toxicity during radiotherapy (RT) for head and neck cancer (HNC). We report the first use of KeraStat^®^ Cream (KC), a topical, keratin-based wound dressing, in patients with HNC receiving RT.

**Methods:**

This pilot study randomized HNC patients treated with definitive or postoperative RT (≥60 Gy) to KC or standard of care (SOC), applied at least twice daily during and for 1-month after RT. Outcomes of interest included adherence to the assigned regimen (at least 10 applications per week of treatment), clinician- and patient-reported RD, and skin-related quality of life.

**Results:**

24 patients were randomized and completed the study. Most patients had stage III-IV disease and oropharynx cancer. Median RT dose was 68 Gy; the bilateral neck was treated in 19 patients, and 18 patients received concurrent chemotherapy. Complete adherence was observed in 7/12 (SOC) vs. 10/12 (KC, *p* = 0.65). Adherence by patient-week was 61/68 versus 64/67, respectively (*p* = 0.20). No differences in RD were observed between groups.

**Conclusion:**

A randomized trial of KC versus SOC in HNC patients treated with RT is feasible with good adherence to study agent. An adequately powered randomized study is warranted to test the efficacy of KC in reducing RD.

## Introduction

Radiotherapy (RT) is an important treatment for head and neck cancer (HNC), but the high burden of acute and late toxicities can significantly impact patient quality of life.^1^ One of the most bothersome and difficult-to-manage physical manifestations of RT injury to normal tissue is acute radiation dermatitis (RD) that usually starts within the first 2–4 weeks of RT. RD usually increases during the course of treatment and persists until approximately 2 weeks after completion of conventionally fractionated RT.^2^ Its early manifestations differ between skin tone types: erythema in patients with lighter skin tones and hyperpigmentation in patients with more pigmented skin types, the latter of which is not well captured in commonly used RD grading scales.^3–6^ Further progression of RD can manifest as dry or moist desquamation (i.e., moderate RD) to oedema, bleeding, ulceration or necrosis (i.e., severe RD).

The major cause of RD is thought to be the gradual decrease in keratinocyte production through cell death in the more radiation-sensitive epidermal basal layer of the stratum basale and also by the associated inflammatory reaction of cytokines and chemokines such as interleukin (IL)-1α, IL-1β, tumour necrosis factor (TNF)-α, IL-6, IL-8, C–C motif chemokine ligand 4 (CCL4), C–X–C motif chemokine ligand 10 (CXCL10) and C–C motif chemokine ligand 2 (CCL2).^7^ This decrease in keratinocyte production and increase in inflammation frequently leads to desquamation, with disruption of the skin’s barrier function.^8–10^ Recent studies of head and neck (HN) RT with or without concurrent chemotherapy/biologic therapy report acute RD rates up to 94% for any grade, up to 80% for grade 2+ and up to 25% for grade 3+.^11–15^ The development of moderate to severe RD can significantly impact both health-related quality of life and the course of treatment. If the skin reaction is severe enough, a treatment break may be recommended that prolongs the course of RT and may result in decreased rates of local tumour control or overall survival.^16,17^ Managing the symptoms associated with RD is important for ensuring the completion of RT in a timely manner, which is particularly critical in the treatment of HNC.^18,19^ Once moderate to severe RD occurs, management generally converts to therapeutic rather than preventive and includes topical burn dressings such as Mepilex dressings or silver sulfadiazine creams.^20,21^

Currently, standard skin care for patients treated with RT includes the use of routine gentle washing of the treated site daily, with or without topical non-fragrant, hypoallergenic emollient moisturisation.^20–24^ Of the multiple topical agents tested for the prevention and/or treatment of RD, few agents have successfully shown a clinically meaningful benefit.^21,25^ While topical corticosteroid therapy may be associated with reduced rates of RD with moist desquamation in breast cancer patients undergoing RT, the evidence is conflicting, and this has not been tested in the context of HN RT which frequently involves higher doses than breast RT.^26–28^ Due to the complex anatomy of HNC, differences in RT planning and delivery, the higher RT doses, and concurrent chemotherapy often applied in HNC, it is difficult to generalise findings from the treatment of other cancers to the treatment of HNC. Additionally, local skin and soft tissue effects of prolonged topical steroid use make this option a problematic alternative.^29^ Further investigations of non-steroidal topical agents to reduce the symptomatic burden of HN RT are needed.

Here, we report the first use of a topical product for RD, KeraStat^®^ Cream (KC), for HNC patients treated with RT. KC is currently the only topical product on the US market that has undergone a full Federal Drug Administration (FDA) panel review and has been cleared for the indication of RD associated with RT. KC is a non-sterile, non-implantable, emulsion-based wound dressing intended to manage the skin toxicity that occurs with radiation treatment. KC uses human hair-derived keratin proteins which *in vitro* studies have shown preferentially polarise undifferentiated macrophages to the anti-inflammatory M2 phenotype and attenuate the pro-inflammatory IL-1α, IL-1β and IL8 responses while upregulating the anti-inflammatory IL33 response.^30,31^ KC is being assessed by the Biomedical Advanced Research and Development Authority (BARDA) under a current contract to serve as a medical countermeasure for ionising radiation injury using an established model of cutaneous radiation injury.^32^ All the ingredients of KC have been used in FDA-approved products, and a biological safety profile has been established for the keratin protein in KC, which has been assessed by the FDA to be a non-irritant with no proclivity for inducing an immune response.^33^ It has also been recently studied in a prospective clinical trial for patients treated with breast RT.^34^ Thus, KC is a promising topical agent to promote wound healing in patients treated with RT at risk of developing RD in various anatomical sites.

Despite the number of studies on the topic, there is extremely limited evidence on the level of adherence to assigned skin care in published clinical trials and even fewer data on skin care adherence in the real world. However, when frequent monitoring and reinforcement of the recommended skin care regimen is conducted as part of a prospective clinical study, adherence has been estimated to be as high as 85–95%.^35,36^ Given the clinical experience and the limited data on this topic, we posited that adherence among the general HNC population receiving RT is somewhat lower than these previously reported values. An understanding of adherence to an assigned skin care regimen in a randomised, open-label clinical trial was warranted to inform future studies of topical interventions for acute RD. In this single-institution, randomised pilot study, we aimed to primarily assess adherence to the study intervention and, secondarily, the cumulative incidence of RD and quality of life outcomes for patients with HNC treated with RT.

## Materials and Methods

### Study design and patient selection

This study was designed as a randomised open-label pilot study to test the feasibility of a randomised trial comparing KC with standard of care (SOC) in HNC patients. Patients were eligible for enrolment if they had a diagnosis of HNC and were scheduled to receive conventionally fractionated RT to the HN with a total dose of at least 60 Gy. Patients had to be able and willing to sign informed consent and to complete adherence, RD-related outcome and skin-related quality of life surveys. Patients with a history of prior HN RT, recent or active use of topical corticosteroids in the region to be treated, scleroderma, active systemic lupus erythematosus requiring systemic medication and those planned for concurrent anti-epidermal growth factor receptor therapy (i.e., cetuximab) were excluded. All patients provided written informed consent prior to registration. Patients were randomised 1:1 to either SOC or KC. This pilot study was designed to confirm the feasibility and defined *a priori* as complete adherence to skin care regimen in at least 60% of patients, with a sample size large enough (e.g., our sample of *n* = 24) to allow an adequate degree of precision in estimating adherence. We reasoned that if this metric was satisfied, then the intervention would be worth pursuing in a future signal-of-efficacy study with a larger sample size. Thus, the primary endpoint was adherence to the assigned topical agent. Secondary endpoints included rates of clinician- and patient-reported acute RD and patient-reported measures of skin-related quality of life.

### Study interventions

Beginning on the first day of RT, all patients were instructed to apply their assigned agent at least twice daily (more frequently was allowed) to the skin of the HN within the treated region. Patients assigned to the KC group were instructed to use KC and no other topical agent. Those assigned to SOC were recommended to select one of a list of commercially available over-the-counter topical agents as listed in Supplemental Table 1. Patients were also recommended to practise daily gentle washing of the treated area with a mild soap.^20^ Any patient who developed moist desquamation or grade 3 + RD was instructed to cease application of the assigned agent on that skin region, and an absorbent wound dressing was placed on the sites of moist desquamation per institutional SOC. The assigned topical agent was continued in the regions of the skin without moist desquamation.

### Radiation treatment characteristics

Patients were treated with radiation according to the institutional SOC using a total dose of 63–70 Gy in 28–35 fractions for definitive therapy and 60–66 Gy in 30–33 daily fractions for postoperative treatment, with treatment volumes as described in detail previously.^37^ Twenty-three patients were treated with volumetric-modulated arc therapy; the one treated with 3D conformal therapy was treated for early-stage glottic squamous cell carcinoma using an opposed lateral beam arrangement. Radiation dosimetric factors for the skin organ at risk were derived from the treatment planning system and defined as a 5-mm-thick rind of tissue contracted from the external contour of the patient.

### Assessments and outcomes

Adherence to the assigned study agent was measured once weekly during treatment and was defined as the self-reported number of applications in the prior 7 days, with at least ten applications being judged as adherent to the treatment regimen. Acute RD was assessed weekly during treatment and at 1 month by the treating radiation oncologist or study staff designee using the National Cancer Institute’s Common Terminology Criteria for Adverse Events (CTCAE) version 5.0 RD scale. At the same time points, patients were asked to complete patient-reported outcome (PRO)-CTCAE version 1.0 measures of radiation skin reaction, skin dryness and itching scales. For each of these scales, the severity scale is comprised of none, mild, moderate, severe and very severe. Skin darkening was measured as a binary outcome (yes or no). Self-reported skin-related quality of life was assessed at these time points using the Dermatology Life Quality Index (DLQI), a ten-item measure graded on a 4-point Likert scale (0–3). This item is scored with a range of 0–30 points, where a higher score indicates a greater skin-related quality of life impairment.^38,39^ All PRO measures were administered on paper by providing the patient the opportunity to complete the measure in the presence of site research staff.

### Statistical analysis

We examined a variety of sociodemographic, medical and cancer treatment-related variables at baseline by treatment group; categorical variables were compared across groups using Fisher’s exact tests for differences in proportions, while continuous variables were compared across groups using Wilcoxon tests for differences in medians.

To examine adherence, we dichotomised patients’ answers regarding cream applications per week: if they reported fewer than ten applications in a given week, they were deemed non-adherent for that week, while those reporting at least ten applications in the given week were deemed adherent for that week. Note that because the question about applications was asked on a ‘per week’ basis and was intended to refer to a full 7 days, we began examining this measure at week 2 for each participant, rather than week 1, since ‘week 1’ often contained fewer than 7 days for most participants.

We report adherence data both at the patient level (i.e., patient was fully adherent if all weeks for that patient were adherent) and by the level of weeks across all patients in a group (i.e., proportion of all patient-weeks that were adherent). For the outcomes derived from clinician responses to the CTCAE RD scale and patients’ responses on the PRO-CTCAE radiation skin reaction scale, we considered both dichotomised versions (scores of 2 or higher on either scale) in reporting cumulative incidences of toxicity, and we also evaluated the original ordinal-scale values reported each week in repeated measures models. Using the intent-to-treat principle, mixed models with a random subject effect were used to characterise the CTCAE RD scores and PRO-CTCAE radiation skin reaction scores over time. Included as predictors were week (treated as a categorical variable to allow for non-monotonicity), treatment assignment (SOC versus KC) and the week-by-treatment interaction. We examined the PRO of skin dryness and itching (PRO-CTCAE scales for dryness and itching) using a similar modelling approach as described above. All analyses were carried out in SAS 9.4. A two-tailed alpha of 0·05 was used throughout.

## Results

### Patient population and treatments

A total of 28 patients were enrolled between July 2020 and January 2022; 24 patients (12 in each group) were randomised and completed the study. The CONSORT flow diagram is displayed in Supplemental Figure 1: four patients were enrolled but did not complete the study due to patient preference (*n* = 2), progression of disease (*n* = 1) and death unrelated to this study (*n* = 1). Baseline patient characteristics are summarised in Table 1. The median age of the sample was 64 years (range 30–78), and 18/24 (75%) of the sample were male. Half (12/24) of the patients self-reported as smokers, and nine patients (37·5%) had diabetes as a comorbidity. The primary cancer site was most frequently the oropharynx (*n* = 11, 46%) followed by the oral cavity (*n* = 7, 29%), larynx (*n* = 5, 21%) and sinonasal (*n* = 1, 4%). Seventeen patients (70·8%) had locally advanced (stage III–IV) disease. Of the baseline characteristics examined, only the T stage distribution differed significantly between the two groups (Fisher’s exact *p* = 0·02)—a substantially higher proportion in the SOC group were classified as T4.

The median RT dose was 68 Gy (range 60–70), median skin V60 was 37·2 cc (range 1·5–107·6) and median skin V70 was 0 cc (range 0–24·9) with no differences between groups (Table 2). RT neck target was bilateral, unilateral and none in 19 (79·2%), 2 (8·3%) and 3 (12·5%) patients, respectively. Concurrent chemotherapy was given in 18 (75%) of the patients, 9 in each group. Bolus was utilised to ensure adequate RT dose to the skin surface in one patient in the SOC group and no patients in the KC group.

### Patient adherence and RD outcomes

Complete adherence to assigned skin care, defined as at least 10 applications of cream per week for the duration of treatment, was observed in 17 of 24 patients: 7 (58%) patients in the SOC group versus 10 (83%) patients in the KC group (*p* = 0·65). When adherence was analysed on a per-week basis, a total of 61 of 68 (90%) adherent patient-weeks occurred in the SOC group versus 64 of 67 (96%) patient-weeks in the KC group (*p* = 0·20). There were no temporal trends to non-adherence: two patients per week were noted to be non-adherent, though the non-adherent individuals varied each week.

The cumulative incidence of CTCAE grade 2 + RD was 58% in the SOC group versus 75% in the KC group (*p* = 0·67) (Table 3). Corresponding cumulative incidences of PRO-CTCAE grade 2+ radiation skin reaction were 83% and 67%, respectively (*p* = 0·64).The proportion of patients with PRO-CTCAE skin darkening at any time point was 67% in the SOC group versus 83% in the KC group (*p* = 0·64). In both groups, 75% of participants reported PRO-CTCAE grade 2 or higher itching at least once over the course of follow-up.

**Figure 1.**
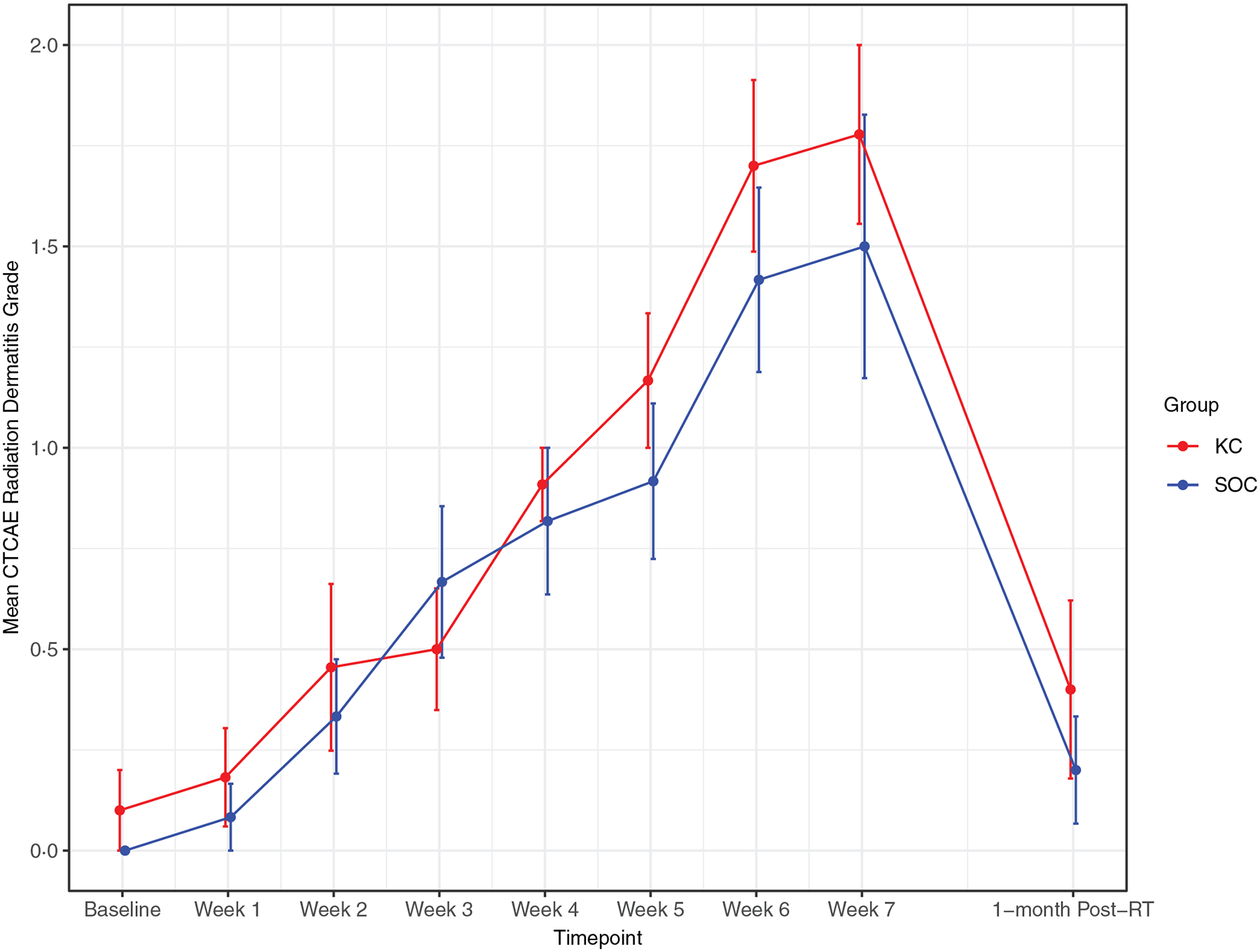
Mean radiation dermatitis CTCAE grade between groups. Error bars indicate standard error.

**Figure 2.**
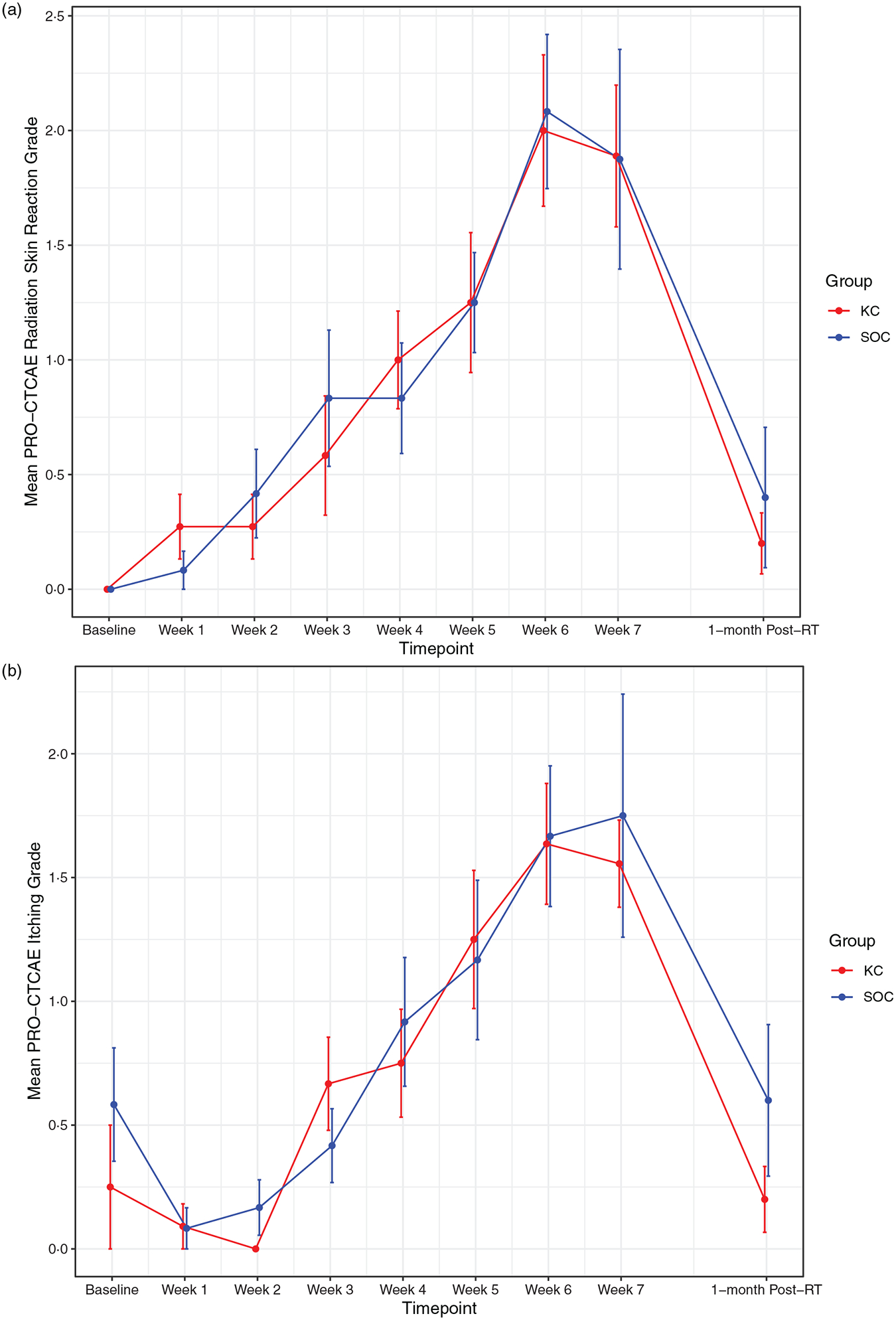

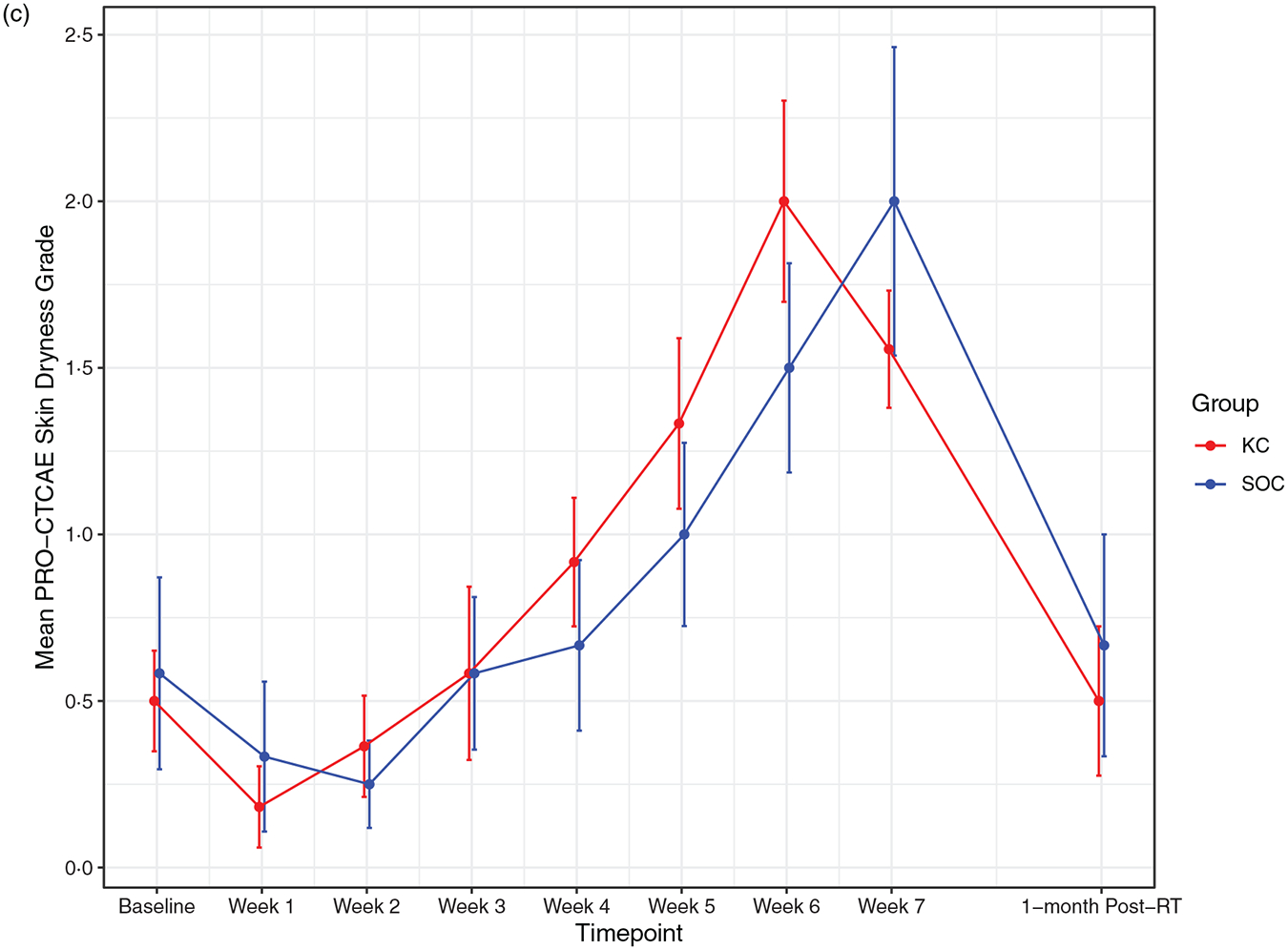
Mean PRO-CTCAE scores for radiation skin reaction (a), itching (b) and skin dryness (c) between groups. Error bars indicate standard error.

**Figure 3.**
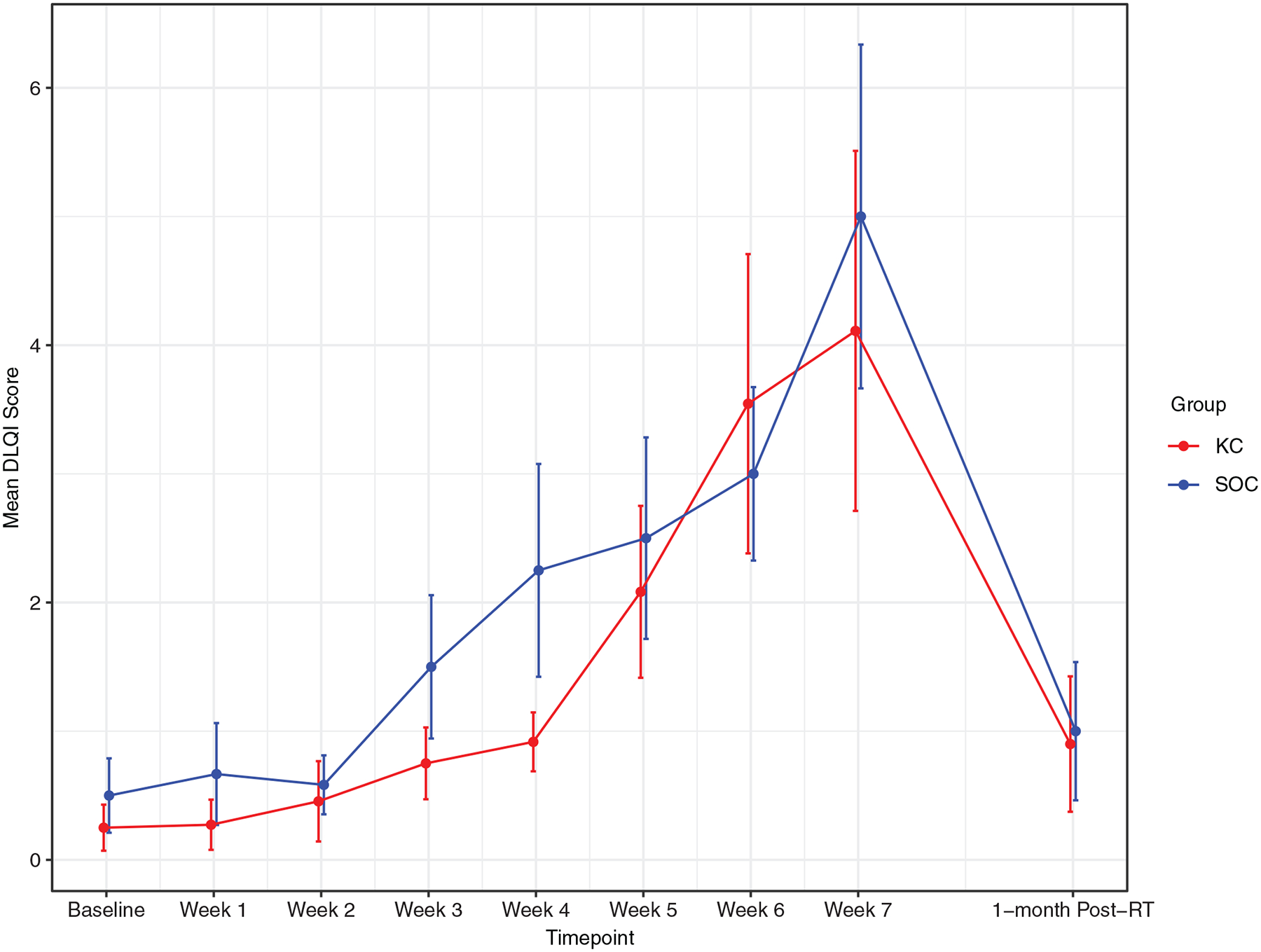
Mean DLQI score between groups. Error bars indicate standard error.

RD outcomes were compared between SOC and KC at end-RT: mean CTCAE RD scores were 1·4 versus 1·8 (*p* = 0·30), mean PRO-CTCAE RD scores were 2·0 versus 2·0 (*p* = 1·0) and DLQI scores were 4·8 versus 4·6 (p = 0·92). The mean CTCAE RD scores at each week (0 through 7) are represented in Figure 1. The mean PRO-CTCAE measures including radiation skin reaction, skin dryness and itching at each week are presented in Figure 2. The mean DLQI scores at each week are shown in Figure 3. For each of the RD scores and for the DLQI, repeated measures analyses considering group, treatment week (as an ordinal variable, 0–7) and the group–week interaction identified a consistent, highly significant (*p* < 0·0001) association between outcome score and treatment week, with significantly and increasingly higher scores over time. This strong effect of week was observed equally in both groups, for none of the outcome variables was the treatment group–week interaction significant. Put differently, in both groups, RD and DLQI scores increased similarly and significantly with each week of treatment. For CTCAE RD, the *p*-value for the treatment group–week interaction was 0·72; for PRO-CTCAE RD, this *p*-value was 0·96; and for the DLQI score, it was 0·78.

### Exploring associations between clinician-rated and patient-reported measures of dermatitis

An exploratory analysis of the agreement between the clinician-rated and patient-reported CTCAE measures of RD was performed using all 182 available assessments where both CTCAE and PRO-CTCAE grades were available. We first used the dichotomous versions of these outcomes to assess agreement. For the 151 assessments where the clinician had not given a score of 2 or higher on the CTCAE scale, 81·5% of the patients also reported a score of less than 2 on the PRO-CTCAE measure. Of the 31 assessments where the clinician had given a score of 2 or higher, 32·3% of the patients did not report a score of 2 or higher. Overall, a total of 38 of 182 assessments (20·9%) were discordant on these dichotomous rankings, resulting in a McNemar’s test *p*-value of 0·0035, suggesting significant discordance. Supplemental Table 2 depicts the distribution of the RD scores reported by both clinicians and patients in the 182 assessments as full ordinal variables (scored from 0 to 4) so that specific concordant/discordant patterns can be examined in more detail.

## Discussion/Conclusion

Acute RD is one of the most common and problematic acute toxicities associated with HN RT. Despite this, there remains a limited selection of topical agents that are effective in the prevention and/or management of moderate to severe RD in the HNC patient population. Though several agents have been investigated over the years, a recent meta-analysis of multiple pharmacologic and non-pharmacologic topical agents found only olive oil to be associated with reduced odds of the development of RD.^40^ The topical approach to mitigation of acute RD has been more extensively studied in patients with breast cancer treated with adjuvant RT, with a consistent theme indicating improvements in dermatitis grade and incidence of moist desquamation using topical corticosteroids.^27,41^ These patients differ greatly from patients with HNC, as they are often treated with lower doses of RT and do not routinely receive concurrent radiosensitising chemotherapy. This may explain why the benefits seen in the breast cancer population are not as clear in trials of corticosteroids for RD in HNC patients.^42,43^ In an effort to improve the quality of life during therapy, as well as maximise RT adherence for patients with HNC, there is a significant need to expand the currently available selection of topical agents for RD.

This study represents the first clinical trial investigating the use of KC in patients with HNC. The first step in understanding the effect of KC on the development of acute RD was to determine patient adherence to the skin care regimen using KC, as well as the feasibility of a randomised comparative clinical trial. The study sample was generally representative of the overall HNC population in terms of stage, primary site and use of concurrent systemic therapy. Complete adherence was numerically higher in the KC group (83%) compared to SOC, and adherence using week as the unit of observation was very high in both groups (90–96%). While these findings are overall favourable, it is worth considering that adherence was self-reported by patients and may be subject to self-reporting bias. Because the application of a topical agent does not lend itself easily to tube count-back or other monitoring methods for adherence, it was not feasible to objectively measure adherence (i.e., by directly monitoring the application). For this reason, the adherence rates observed in this study should be interpreted with caution. Treatment adherence is heterogeneously reported and difficult to interpret in the medical literature, but a standardised classification and framework for non-adherence reporting do exist.^44,45^ Future studies of KC for RD must identify the following aspects of non-adherence: the type (e.g., deviation from regimen, temporary or permanent discontinuation), the decision-maker responsible (e.g., investigator, other medical professional or patient), the underlying reasons for non-adherence and the timing.^45^

Although not sufficiently powered to measure efficacy, the observed cumulative incidence of CTCAE grade 2 + RD was consistent with prior studies of HN RT. It should be noted that the heterogeneity with which acute RD is reported, even among trials reporting CTCAE dermatitis grade, makes it difficult to determine an expected incidence based on the available literature.^13,14^ Patient-reported incidences of moderate to severe radiation skin reaction, skin darkening and itching appeared similar to expected rates. All outcomes followed the expected time course with gradual development during treatment, reaching a peak towards the end of treatment and improving by the 1-month follow-up visit. Importantly, we utilised multiple criteria to assess the severity of RD during and after RT: CTCAE score, PRO-CTCAE measures of radiation skin reaction, hyperpigmentation and itching, as well as the DLQI. Considering the variable manifestations of RD that may not be well captured using a singular clinician-rated scoring system developed primarily for patients with lighter phototypes, and variation in rates of acute RD in patients with different skin types, a diverse battery of clinician-rated and PRO measures is needed to ensure accurate and equitable measurement of acute RD across a diverse patient population.^3,46^ While skin phototype was not collected at baseline in the present study, a future clinical trial will assess patient-reported Fitzpatrick skin type to better understand and account for the interaction between skin type, acute RD and treatment effect.^47,48^

Another important finding of this study is that exploratory analyses of agreement between clinician-rated and patient-reported measures of acute RD identified substantial discordance. Using the dichotomous variable grade 2 or higher by CTCAE or PRO-CTCAE (moderate to severe RD), approximately one in five patients reported moderate to severe ‘radiation skin reaction’ after being assigned none to mild RD by clinician-rated CTCAE criteria. Conversely, about one in three patients reported none to mild radiation skin reaction by PRO-CTCAE after being assigned CTCAE RD grade 2 or higher. This discordance is perhaps not surprising given that a score of 2 or higher on the CTCAE RD scale depends on objective observations of either brisk erythema or the more clinically relevant finding of moist desquamation. In contrast, the analogous PRO-CTCAE measure of radiation skin reaction does not include objective findings like desquamation but instead relies on subjective assessments of the skin reaction’s severity. These findings are in agreement with prior studies that clinician-rated dermatitis outcomes show low to moderate correlation with PRO and that each individual outcome measure best correlates with other measures within its category (clinician-rated or patient-reported).^49,50^ This finding is of interest to future study designs and highlights the importance of comprehensive measure selection to adequately capture clinically relevant outcomes (i.e., those that prompt a change in management) while maintaining a focus on the patient’s perception of severity. The development of moist desquamation is often a major contributor to pain and discomfort in patients with acute RD. It often warrants additional management or at least a change in the prior ‘preventive’ topical management. In previous studies, clinician-rated and patient-reported measures of moist desquamation are associated with reasonably high levels of agreement, indicating this dichotomous endpoint could be assessed either by objective clinician examination or patient report.^51,52^ Since the development of moist desquamation is such a clinically meaningful event for patients, outcome measures focused on the cumulative incidence of moist desquamation rather than CTCAE grade 2 + RD (which includes patchy moist desquamation, moderate/brisk erythema or moderate oedema) may more reliably identify topical agents with clinical benefit.^27^

This study was not powered to detect a difference in the cumulative incidence of RD between groups, so the findings regarding RD should be interpreted with caution. Another limitation is that we allowed patients randomised to the SOC group to select the topical agent of their choice from a list of multiple commercially available emollients and ointments that are commonly used during RT. While this may have introduced heterogeneity to the control group, this decision was pragmatic—to approximate real-world practice by considering one of many currently available SOC agents. In a future randomised controlled trial, the control group may be limited to a single emollient such as Eucerin or Sorbolene, as has been used in prior studies.^27,53^ The study is also limited by the heterogeneous patient population; further limiting the selection of patients to those at high risk of RD (e.g., patients planned for concurrent chemoradiotherapy and/or bilateral neck treatment) may improve the chances of detecting a benefit in a larger study. A skin dosimetric threshold may be useful for the selection of patients at high risk of RD, though the optimal dose-volume parameter and threshold are not clear. Another limitation is the lack of blinding; the colour and texture of KC make it difficult to find a matched control agent, so it was not feasible to blind patients to the intervention. While blinding of the RD rater was outside of the scope of this pilot study, a single-blind methodology, ideally with a central review of dermatitis outcomes using photographic documentation by multiple blinded assessors, would substantially limit the risk of detection bias. Another limitation of this study is the lack of assessments in the acute post-RT period that may have resulted in missed RD events. This study was designed to minimise patient burden by coupling study assessments with routine clinic visits. To accurately describe the incidence, severity and duration of RD, weekly assessments for at least 4 weeks after RT would be optimal. Regarding assessment methods and frequency, PRO survey results may be subject to bias from varying levels of participant health literacy and may also be affected by frequent sampling (i.e., weekly PRO assessments). These factors may be ameliorated in future trials by randomisation, collection of baseline health literacy and the use of repeated measures analyses.

In conclusion, this randomised pilot study of KC versus routine skin care for acute RD in HNC patients demonstrates excellent adherence and supports the feasibility of a larger randomised study comparing KC versus SOC. In order to determine the clinical benefit of KC to reduce the incidence and severity of acute RD, a future well-powered comparative effectiveness clinical trial is needed.

## Supplementary Material

Supplementary materials

## Figures and Tables

**Table 1.** Patient baseline characteristics

	SOC	KeraStat^®^	*p*-Value
Median age in years (range)	64·5 (47–78)	63·5 (30–77)	0·61
Gender			0·16
Female	5 (41·7)	1 (8·3)	
Male	7 (58·3)	11 (91·7)	
Race			1
White	11 (91·7)	10 (83·3)	
Black	1 (8·3)	1 (8·3)	
Other	0 (0)	0 (0)	
Non-Hispanic ethnicity	12 (100)	12 (100)	1
Smoking history			0·41
Ever smoker	7 (58·3)	5 (41·7)	
Never smoker	5 (41·7)	7 (58·3)	
Median pack-years among smokers (range)	21 (2–140)	150 (100–800)	0·17
Primary cancer site			0·32
Oropharynx	7 (58·3)	4 (33·3)	
Oral cavity	4 (33·3)	3 (25·0)	
Larynx	1 (8·3)	4 (33·3)	
Nasal/paranasal	0 (0)	1 (8·3)	
T stage			0·02
T0	0 (0)	1 (8·3)	
T1	3 (25·0)	2 (16·7)	
T2	2 (16·7)	4 (33·3)	
T3	0 (0)	4 (33·3)	
T4	7 (58·3)	1 (8·3)	
N stage			0·12
N0	2 (16·7)	3 (25·0)	
N1	4 (33·3)	1 (8·3)	
N2	6 (50·0)	4 (33·3)	
N3	0 (0)	4 (33·3)	
Stage			0·31
I	1 (8·3)	1 (8·3)	
II	2 (16·7)	3 (25·0)	
III	5 (41·7)	1 (8·3)	
IV	4 (33·3)	7 (58·3)	
Median BMI (range)	28·7 (23·8–40·7)	26·9 (19·8–36·8)	0·37
Diabetes			1·0
Yes	4 (33·3)	5 (41·7)	
No	8 (66·7)	7 (58·3)	
Diabetes with insulin dependence	0 (0)	3 (25·0)	0·28
Diabetes with insulin independence	4 (33·0)	1 (8·3)	0·32

Group differences in medians tested with a Wilcoxon test; differences in proportions tested with Fisher’s exact test.

**Table 2.** Oncologic treatment characteristics

	SOC	KeraStat^®^	*p*-Value
RT dose, median (range)	70 (60–70)	66 (60–70)	0·47
RT number of fractions, median (range)	35 (29–35)	33 (28–35)	0·32
Surgery			0·67
Yes	3 (25·0)	5 (41·7)	
No	9 (75·0)	7 (58·3)	
RT neck target			
Bilateral	9 (75·0)	10 (83·3)	0·59
Ipsilateral	2 (16·7)	0 (0)	
None	1 (8·3)	2 (16·7)	
Bolus used			
Yes	1 (8·3)	0 (0)	1·0
No	11 (91·7)	12 (100)	
Concurrent chemotherapy			
Yes	9 (75·0)	9 (75·0)	1·0
No	3 (25·0)	3 (25·0)	
Skin V40* in cm^3^, median (range)	168·2 (30·1–311·4)	199·5 (30·2–254·7)	0·77
Skin V50* in cm^3^, median (range)	114·6 (16·4–220·7)	130·4 (12·1–186·6)	0·91
Skin V60* in cm^3^, median (range)	37·7 (5·6–107·6)	37·2 (1·5–89·9)	0·59
Skin V70* in cm^3^, median (range)	0·01 (0–24·9)	0 (0–5·2)	0·11

Group differences in medians tested with a Wilcoxon test; differences in proportions tested with Fisher’s exact test.

* The skin organ at risk structure was delineated using a rind of thickness 5 mm contracted from the external contour of the patient. The volume receiving at least x Gy (Vx) is expressed as an absolute volume in cm^3^.

**Table 3.** Cumulative incidence of clinician-rated and patient-reported radiation dermatitis outcomes

	SOC	KeraStat^®^	*p*-Value
CTCAE grade 2 + radiation dermatitis	7 (58)	9 (75)	0·67
PRO-CTCAE grade 2 + radiation skin reaction	10 (83)	8 (67)	0·64
PRO-CTCAE grade 2 + skin dryness	8 (67)	10 (83)	0·64
PRO-CTCAE grade 2 + itching	9 (75)	9 (75)	1·0
PRO-CTCAE skin darkening	8 (67)	10 (83)	0·64

Group differences in medians tested with a Wilcoxon test; differences in proportions tested with Fisher’s exact test.

## Data Availability

Research data are stored in an institutional repository and will be shared upon request to the corresponding author.
