# Supplementary material for: Keratin-based topical cream for radiation dermatitis during head and neck radiotherapy: a randomised, open-label pilot study
Source: J Radiother Pract. Author manuscript; Available in PMC 2024 Oct 3. (PMC11449460; doi:10.1017/s1460396924000037)
Supplement: Supplementary materials [file NIHMS1990624-supplement-Supplementary_materials.docx]

Supplemental Figure 1: CONSORT Flow Diagram


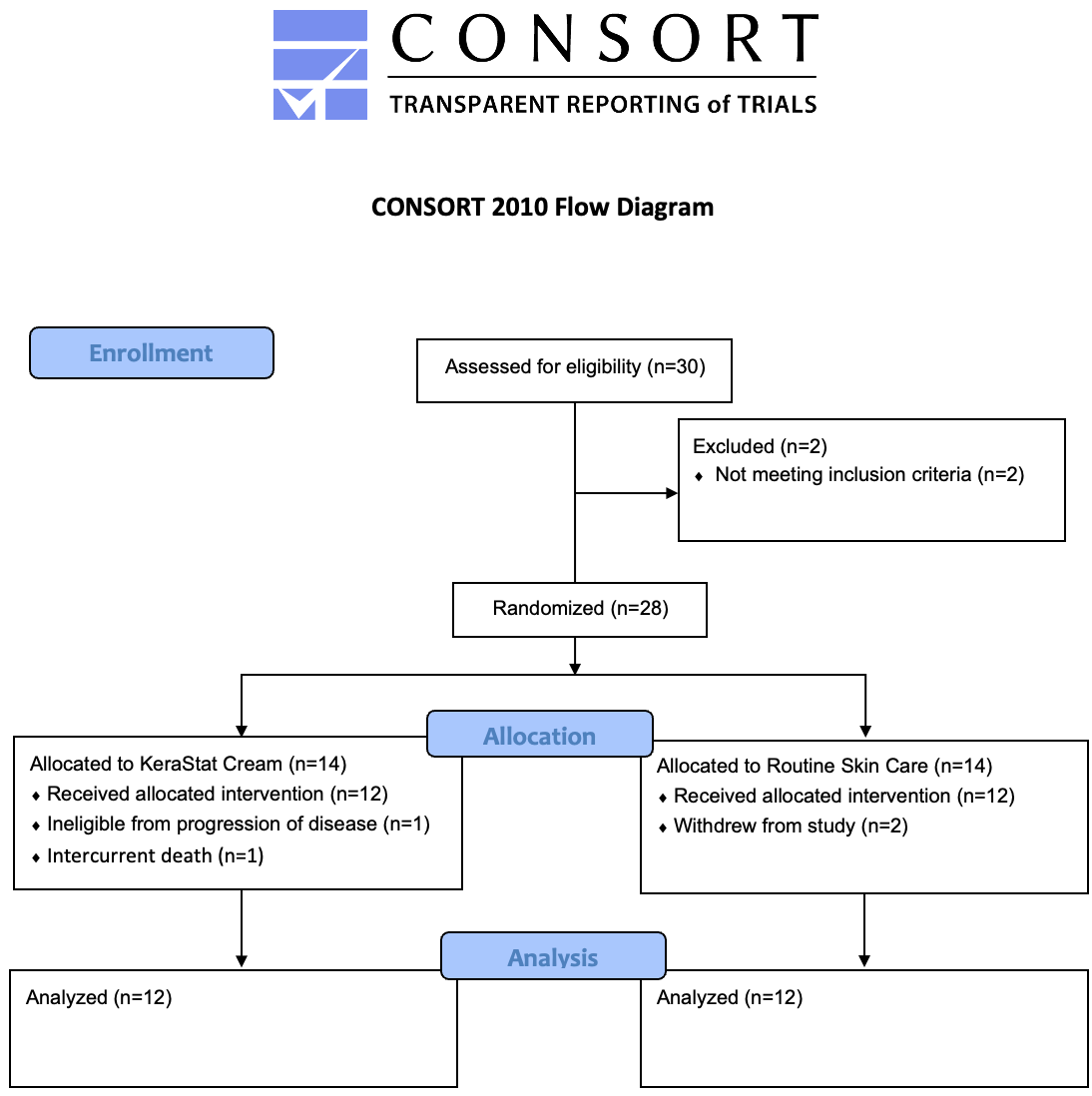


Supplemental Table 1: Options for topical agents utilized in the standard of care group

| Petroleum-based ointments (Aquaphor®, Balmex®, EltaMD®) |
| --- |
| Eucerin® |
| Lubriderm® |
| Aveeno® |
| Calendula-containing creams or gels |
| Cetaphil® |
| CeraVe® |

Supplemental Table 2: Agreement between the CTCAE and PRO-CTCAE measures of radiation dermatitis. Data are presented as count (percent of total).

| **CTCAE Radiation Dermatitis Grade** | **PRO-CTCAE Radiation Skin Reaction Grade** | | | | | |
| --- | --- | --- | --- | --- | --- | --- |
|  | **0** | **1** | **2** | **3** | **4** | **Total** |
| **0** | 59 (33.3) | 13 (7.3) | 6 (3.4) | 1 (0.6) | 0 (0) | 79 (44.6) |
| **1** | 26 (14.7) | 22 (12.4) | 17 (9.6) | 2 (1.1) | 0 (0) | 67 (37.9) |
| **2** | 2 (1.1) | 8 (4.5) | 10 (5.7) | 4 (2.3) | 4 (2.3) | 28 (15.8) |
| **3** | 0 (0) | 0 (0) | 1 (0.6) | 1 (0.6) | 1 (0.6) | 3 (1.7) |
| **4** | 0 (0) | 0 (0) | 0 (0) | 0 (0) | 0 (0) | 0 (0) |
| **Total** | 87 (49.1) | 43 (24.3) | 34 (19.2) | 8 (4.5) | 5 (2.8) | 177 (100) |
